# Assessment of Metal Contamination in Water of Freshwater Aquaculture Farms from a South Asian Tropical Coastal Area

**DOI:** 10.3390/toxics10090536

**Published:** 2022-09-14

**Authors:** Mohammad Belal Hossain, Md. Robel Miazie, As-Ad Ujjaman Nur, Shyamal Kumar Paul, Muhammad Abu Bakar, Bilal Ahamad Paray, Takaomi Arai

**Affiliations:** 1 Department of Fisheries and Marine Science, Noakhali Science and Technology University, Noakhali 3814, Bangladesh; 2 School of Engineering and Built Environment, Griffith University, Brisbane, QLD 4111, Australia; 3 Bangladesh Council of Scientific and Industrial Lab (BCSIR), Chittagong 4220, Bangladesh; 4 Department of Zoology, College of Science, King Saud University, P.O. Box 2455, Riyadh 11451, Saudi Arabia; 5 Environmental and Life Sciences Programme, Faculty of Science, Universiti Brunei Darussalam, Jalan Tungku Link, Gadong BE1410, Brunei

**Keywords:** heavy metal, surface water, aquaculture farms, central coast

## Abstract

Heavy metal accumulation in aquaculture farms has become a major problem due to the widespread use of artificial feed to enhance fish productivity. To estimate the contamination level and identify metal sources, we investigated the amounts of seven heavy metals (Cu, Zn, Pb, Cd, Cr, Ni, and Mn) in the surface water of commercial fresh water aquaculture farms in a south Asian tropical coastal area. Atomic absorption spectrometry (AAS) was used to analyze 36 water samples from 12 commercial fish farms. The results demonstrated that the range of three heavy metals were detected in a decreasing order of Mn (0.0574–0.4100 mg.L^−1^) > Zn (0.0125–0.3250 mg.L^−1^) > Cu (0.0275–0.085 mg.L^−1^). In all samples, the remaining four heavy metals (Pb, Cd, Cr, and Ni) were below the detectable level (BDL). Except for Mn, the amounts of the metals examined were below WHO and USEPA guideline values. According to the findings, the levels were found to be safe for drinking, agriculture production, and aquaculture. There was no significant correlation (*p* > 0.05) between heavy metal concentrations and water quality parameters, indicating that pollution came from diverse sources and that no single factor was controlling their levels. Furthermore, Analysis of Variance (ANOVA) revealed no significant differences in the mean metal values among the fish farms (*p* > 0.05). Multivariate analyses (CA and PCA) demonstrated the association and sources of metal in the study area. Although metal levels were not beyond the threshold limit, it is recommended that suitable measures and continuous monitoring should be undertaken to reduce heavy metal pollution in aquaculture farms and prevent water quality degradation.

## 1. Introduction

Bangladesh has vast and extensive water resources scattered throughout the country in the form of fields, natural depressions such as haors and beels, lakes, canals, reticulated rivers, and estuaries, covering an area of around 4.73 million ha. The total area of farms in Bangladesh is estimated to be 0.39 million ha, with 1.9 million metric tonnes (MT) of fish produced annually [1]. Aquaculture is a significant socioeconomic activity, particularly for rural areas, which contributes to livelihoods, household food security, and poverty reduction through revenue generation, employment, and local and international trade [2,3]. The country is fifth in the world in aquaculture production, contributing 56.24 percent of total fish production according to the FAO study “The State of World Fisheries and Aquaculture 2018” [1].

Recently, heavy metal contamination in the aquatic environment has received global concern for its toxicity, abundance and non-degradable nature, as well as successive accumulation in tropic levels, thus causing lethal biological effects [4,5,6]. Aquatic ecosystems can be contaminated by the accumulation of heavy metals through emissions from the rapid expansion of industrial areas, mine tailings, untreated waste seepages containing toxic metals as well as metal chelates from different industries, e.g., tanneries, steel plants, battery industries, thermal power plants, sewage sludge, application of heavy metal containing fertilizers, pesticides, insecticides, herbicides in agriculture, coal combustion residues, spillage of petroleum and atmospheric deposition [7,8,9]. Heavy metal pollution can deteriorate water quality and a pose threat to human beings as well as aquatic biodiversity [10,11]. Typically, its concentrations in aquatic ecosystems are checked by measuring its concentration in water, sediment and aquatic organisms [12], which generally exist in low levels in water and attain considerable concentrations in sediments and organisms [13].

The most pressing concern in aquaculture sustainability is water quality. As fish swim, relax, defecate, and grow in water, a sustainable and successful aquaculture operation relies on the availability and continual supply of good quality waters. As a result, aquaculture farms are frequently found along river banks or in coastal locations, as these waters often provide optimal conditions for both fresh and salt water aquaculture. However, due to rapid population increase and uncontrolled development in many coastal regions, the aquaculture industry is increasingly threatened by water contamination. These areas get large human inputs from metropolitan centres, tourists, and businesses located along the coasts [10,11]. Heavy metal pollution in coastal areas has been identified as a severe contaminant, as these pollutants in the aquatic environment can harm aquatic life and endanger fish [12]. Aquaculture, which draws water from the river, estuary, and coastal area, is thus vulnerable to pollution from the outside, and the produce (fish) may pose a health concern if consumed.

Heavy metal contaminations in estuarine and coastal areas have been documented as a serious environmental concern [14], as these areas obtain substantial anthropogenic inputs of heavy metals equally from point and nonpoint upstream sources [15]. Lower landmass of coastal areas faces many natural catastrophes almost in every year and thus sea water containing heavy metal intrusion occurs when cyclones or tidal flashes strike, creating a great problem for aquaculture. Various noxious diseases of fish (e.g., edema of eyelids, tumor, congestion of nasal mucous membranes and pharynx, stuffiness of the head and gastrointestinal, muscular, reproductive, neurological and genetic malfunctions etc.) and humans (e.g., cancer, reduced immune function, Parkinson’s disease, neurological and physiological disorders including kidney failure, etc.) caused by some of these heavy metals have been recorded [16,17,18]. Therefore, determining the concentration of heavy metals in the water column is very essential for environmental and human health safety assessment.

Several studies have been conducted on metal levels and its risk assessment in cultured fishes and fish feed of aquaculture farms from Bangladesh [19,20]. Though the ultimate results of excessive heavy metal discharge, accumulation and consumption are known [21,22], research regarding heavy metal concentrations in the waters of freshwater aquaculture farms from the central coast of Bangladesh is scant. Poor management of aquafarms due to lack of authentic information and the levels of pollutants in fish can cause a potential risk to humans who consume them. Therefore, this study was conducted in this area to evaluate the heavy metal contamination level and to identify the sources in the surface water of commercial aquaculture farms.

## 2. Materials and Methods

### 2.1. Study Sites and Water Sampling Method

The study area (Noakhali and Laksmipur) is located between latitude 22°07′ and 23°08′ N and longitude 90°53′ and 91°27′ E along the northern Bay of Bengal coast. These are the two coastal districts in Bangladesh which cover almost the whole central coastal area. As the area is located on the tidal floodplain of the Meghna River delta, it is fertilized by silt deposits from the Meghna estuary. Therefore, the central coast is characterized by flat land and low relief with alluvial soil. The area is strongly influenced by the nearby climatic condition of Bay of Bengal, being more pronounced during the monsoon season. The climate is tropical in the central coastal area of Bangladesh, which is considered to be Aw according to the Köppen-Geiger climate classification. The average annual temperature is 25.2 °C and the rainfall is about 2218 mm annually. The region was selected for the study as there were many commercial fish farms established recently that have fertile land for aquaculture. These districts contribute much of the fresh water aquaculture production in Bangladesh.

**Figure 1:**
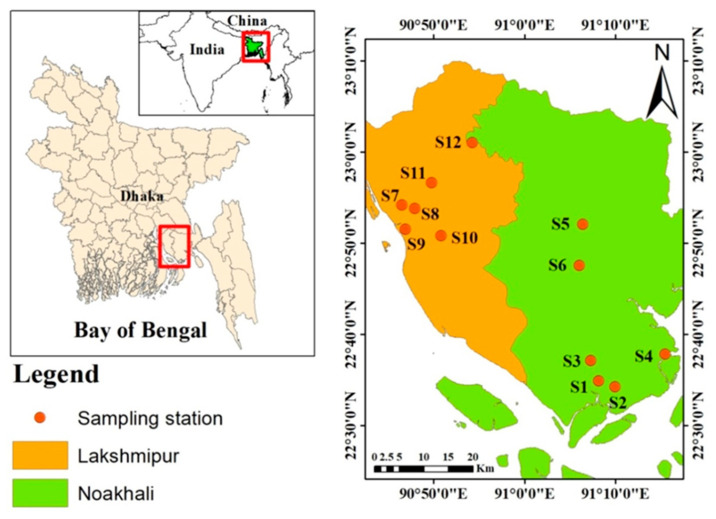
Location of sampling stations (stations 1–6 were in Noakhali and 7–12 were in Lakshmipur district).

A total 36 water samples were collected in March, 2018 from 12 commercial aquaculture farms, each having three replicas (Figure 1). The samples were collected from a depth of 1 foot below the surface using plastic bottles (250 mL) and preserved with 5% nitric acid (HNO_3_) to prevent the decay of heavy metals and also to avoid precipitation. The samples were then placed in an ice-box for transfer to the laboratory and stored at 4 °C until further analysis. Water quality parameters were collected on the spot using various portable instruments. Temperature (°C) and pH were determined by using a microprocessor pH meter (Model No. HI 98139, HANNA Instruments Ltd., Steinbeisstraße, Germany). Salinity (ppt) was determined by a portable Refractometer (model: EXTECH RF20). DO (mg/L) was determined by a digital DO meter (Model No. HI 98139, HANNA Instruments Ltd., Steinbeisstraße, Germany).

### 2.2. Metal Concentration Assessment and Quality Control

The procedure for analyzing heavy metal concentrations in the surface water and sediment samples was carried out following the USEPA method [22]. Briefly, the collected water samples were filtered through a Millipore filtration assembly, using a 0.45 mm membrane filter. For the analysis of total heavy metals (dissolved and suspended), water (200 mL) samples were digested with 5 mL of di-acid mixture (HNO3:HClO4: 9:4 ratio) on a hot plate at 130 °C until the volume came to about 25–30 mL and reached a light color. The addition of HNO_3_ and boiling were repeated until solution became light colored or clear. After cooling, the volume was increased to the desired level with DIW passing through the Whatman 0.42 µm filter paper and made up the volume to 50 mL by double distilled water for the analysis of seven heavy metals viz. Cu, Zn, Pb, Cd, Cr, Ni and Mn using an atomic absorption spectrophotometer (AAS) (Model: AA-700, SHIMADZU, Kyoto, Japan) [23].

Quality assurance and quality control were strictly maintained following United States Environmental Protection Agency (USEPA) guidelines to ensure the accuracy of the analysis. Certified reference material, a CRM 320 (Merck KGaA, Darmstadt, Germany) was employed to provide the validation of data and precision of analytical technique. Ultra-pure HNO_3_ was used for sample digestion. The instrument calibration standards were made by diluting the standard (1000 ppm) supplied by Sigma-Aldrich, Switzerland. The results were expressed as mg.L^−1^ for water samples. De-ionized ultrapure water was used for the experimental procedure. All glassware and containers were cleaned by 20% nitric acid and finally rinsed with de-ionized ultrapure water several times and oven-dried prior to use. The relative standard deviation (RSD) was greater than 5% for all tests, and the recovery rate of the analytical results of metals ranged from 73% to 109%. The limit of detection varied from 0.02 (Cd) to 1.4 (Pb) based on the metals.

### 2.3. Statistical Analysis

The obtained data were statistically analyzed and graphically presented using the Statistical Package for Social Science (SPSS) (version 25) and PAST software, while ArcGIS version 10.3 was used for map plotting. The means and standard deviations of the metal concentration in water and other quality parameters were calculated. Multivariate and univariate statistical analysis such as Pearson correlation matrix (PCM), hierarchical cluster analysis (HCA), and principal component analysis (PCA) were conducted to identify the sources of metals. Analysis of variance (ANOVA) was carried out to test whether there was significant variation of metals among the farms.

## 3. Results and Discussion

### 3.1. Water Quality Parameters

Collected water samples exhibited an alkaline pH which ranged from 7.6 to 8.9 with an overall mean of 8.36 (Table 1). No significant difference was marked in the observed pH of Noakhali and Lakshmipur district and its variation due to change in sampling stations was also insignificant. The observed values were well within the safe limit for aquaculture [24]. A similar trend was also observed in case of salinity that ranged from 0.5 to 1.7 ppt with an average of 0.94 ppt (Table 1). Salinity is considered as the foremost driving factor that affects the density and growth of aquatic organisms [25]. Contradictory results were found in DO levels of the two sampling districts which ranged from 3.5 to 5.39 mg.L^−1^, with an overall mean of 4.60 mg.L^−1^ (Table 1). A significant difference was noted in the observed DO of the Noakhali and Lakshmipur district, where the farms of the Noakhali district had less DO than Lakshmipur. DO levels between 3.0–5.0 ppm in farms are recorded as unproductive, and for good production it should be above 5.0 ppm [26].

### 3.2. Metal Concentration in Water

The presence of three heavy metals, Cu, Zn and Mn were detected in all the samples ranging from 0.028–0.085, 0.013–0.325 and 0.057–0.41 mg.L^−1^ (Table 2), respectively. However, the presence of four toxic heavy metals, Pb, Cd, Cr and Ni were below the detectable level (BDL) in all the samples. An analysis of Variance (ANOVA) was performed to detect the significant differences in the mean value of heavy metal concentration among the water samples of commercial aquaculture farms (Table 2). The ANOVA result showed that the mean heavy metal concentration varied among the twelve selected commercial farms. However, there was no significant variation in the mean concentration level (*p* > 0.05).

The average concentration of the studied metals in the surface water followed a decreasing order of Mn > Zn > Cu. The concentrations of Cu and Zn, however, were within the safe limit for drinking as well as for crop production and aquaculture. The Mn level was slightly higher than the recommended limit set by USEPA and WHO [27]. No industrial activities were found around the sampling sites. The possible sources of heavy metal might be the commercial feed of fish or natural weathering.

The heavy metal concentrations in water of the commercial fish farm were compared with other selected farms and rivers (Table 3). Pb, Cd and Cr were found in the water samples from different farms and rivers at the Khulna-Satkhira region, Bangladesh [26], which was found below the detection level in the present study. Bhuyan et al. [27] found the concentration of metals in the water of Meghna river below the safe limit of the drinking water standards of the World Health Organization (WHO) [28] and the European Union (EU) [29]. Rahman et al. [30] found that the amount of Cu was less than Zn in the sediment and plant samples from the ship breaking area of the Sitakunda coast in Bangladesh, which supports the present findings.

### 3.3. Sources Identification

In order to assess the behavior of heavy metals in the study area, various statistical analyses (PCM, HCA, and PCA) were performed in this investigation. PCM was performed to measure the degree of correlation among heavy metals. No significant correlation was observed among the physico-chemical properties (pH, salinity and DO) and the metals studied in the water of commercial aquaculture farms of greater Noakhali (Table 4), which supports the findings of Kar et al. [33]. As there is no correlation among the elements, the metals are therefore not controlled by a single factor [34].

**Figure 2:**
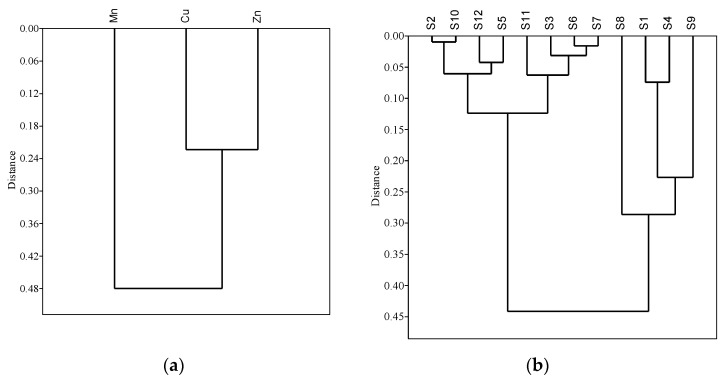
Hierarchical cluster analysis among the selected heavy metals (**a**) and sampling sites (**b**) in the water of commercial farms of the Noakhali and Lakshmipur district; distance metrics are based on the Euclidean single linkage method.

To group the similar selected heavy metals and sampling sites of commercial aquaculture farms of greater Noakhali, an HCA was executed. The analyzed HCA rendered a dendrogram (Figure 2a) where all three metals in the study area were grouped into two groups. Cluster 1 contained Mn only, which was identified as a low to moderate concentration element [35]. Cluster 2 included Cu and Zn which indicated that the farms contained a comparatively higher amount of Cu and Zn.

Similarly, spatial HCA rendered a dendrogram (Figure 2b) where all 12 sampling stations of Noakhali and Lakshmipur were grouped into three clusters. Stations contained in cluster 1 (S2, S10, S12 and S5) were located in low or low to moderate pollution regions. Cluster 2 (S11, S3, S6 and S7) stations were in moderate to high pollution regions [36], whereas the rest of the sampling sites forming cluster 3 (S8, S1, S4 and S9) were in comparatively high pollution regions.

A principal component analysis (PCA) was performed on the normalized data to compare composition patterns. Table 5 summarizes the results of PCA with eigenvalue, variance of 100% and cumulative variance for each factor. Two principal components (PCs) with eigenvalues > 1 were chosen as a consideration. The highest eigenvalue specifies how the data spread, thus the highest eigenvalue is regarded as the principal component.

**Figure 3:**
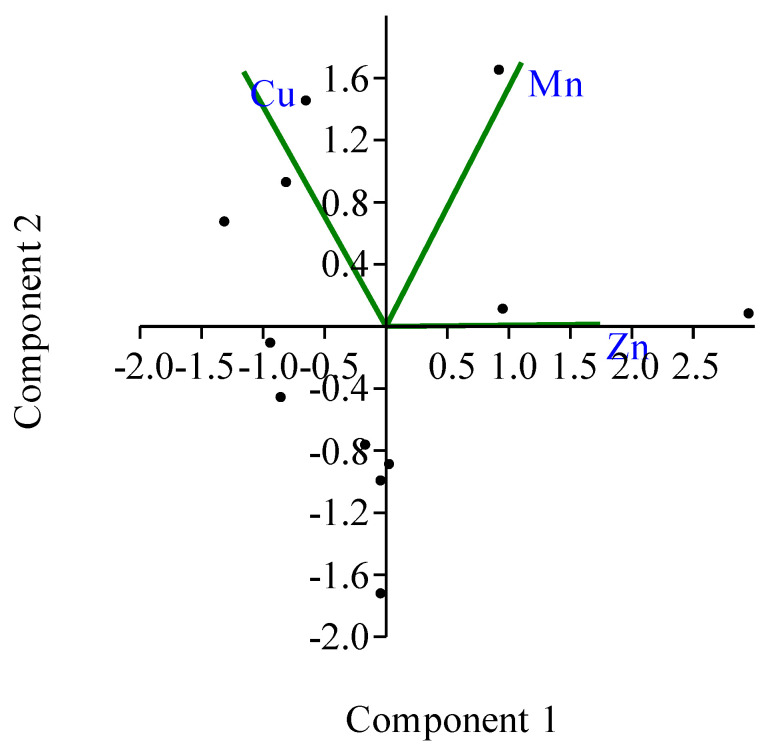
PCA of studied metals in farms water of commercial fish farm of the central coast, Bangladesh.

Principal component (PC1), with the highest eigenvalue of 1.357, is the dominant component. PC1 contributes to 45.229% of the total variance, having a high loading on Zn (R = 0.858). PC2 accounted for 35.527% of the total variance, with moderate positive loadings (>0.50) of Cu and Mn and revealed an eigenvalue of 1.066 (Figure 3). High concentrations of Cu and Mn were possibly caused by anthropogenic inputs from formulated fish feed [37].

## 4. Conclusions

Heavy metal (Cu, Zn, Pb, Cd, Cr, Ni and Mn) concentration in the surface water of the commercial aquaculture farms in Noakhali and Lakshmipur district was measured. The results showed that the mean concentration of the metals in surface water decreased in the following order: Mn > Zn > Cu. There was no strong correlation found among the elements, indicating diverse sources of pollution. From the results, it can be stated that the water of aquatic farms was suitable for aquaculture based on the overall status of heavy metal concentrations in water. Only one metal, Mn, was discovered to be over the recommended levels. It is possible that high levels of Mn were discharged from formulated fish feed. Though some of the detected heavy metals are advantageous for humans and plants up to a certain limit, they would be harmful beyond that limit. The implementation of suitable measures to control the heavy metal concentration in the water of the aquaculture farms is suggested to avoid further deterioration of the water quality.

## Figures and Tables

**Table 1:** Major water quality parameters in commercial aquaculture farms of the central coast, Bangladesh.

St. No.	S_1_	S_2_	S_3_	S_4_	S_5_	S_6_	S_7_	S_8_	S_9_	S_10_	S_11_	S_12_	Mean ± SD
pH	7.6	8.7	8.3	8.8	8.9	8.5	8.23	8.40	8.25	8.48	7.90	8.30	8.36 ± 0.36
Salinity	0.9	1.7	0.9	1.2	0.6	0.7	1.0	1.1	1.2	0.9	0.6	0.5	0.94 ± 0.33
DO	3.55	3.67	4.78	4.80	4.05	3.50	5.39	4.85	5.12	5.30	5.09	5.14	4.60 ± 0.71

**Table 2:** Mean of heavy metals concentration (mg.L^−1^) in water of commercial aquaculture farms of central coast, Bangladesh (mean ± SD).

St.	S_1_	S_2_	S_3_	S_4_	S_5_	S_6_	S_7_	S_8_	S_9_	S_10_	S_11_	S_12_
Cu	0.043 ± 0.003	0.033 ± 0.003	0.085 ± 0.013	0.077 ± 0.01	0.075 ± 0.018	0.055 ± 0.012	0.065 ± 0.01	0.05 ± 0.007	0.038 ± 0.001	0.04 ± 0.009	0.028 ± 0.005	0.033 ± 0.005
Zn	0.113 ± 0.009	0.022 ± 0.005	0.042 ± 0.005	0.015 ± 0.01	0.025 ± 0.008	0.013 ± 0.002	0.033 ± 0.013	0.033 ± 0.004	0.325 ± 0.005	0.033 ± 0.006	0.05 ± 0.008	0.022 ± 0.002
Mn	0.235 ± 0.008	0.133 ± 0.01	0.107 ± 0.002	0.252 ± 0.006	0.19 ± 0.010	0.098 ± 0.007	0.097 ± 0.01	0.41 ± 0.100	0.253 ± 0.013	0.128 ± 0.009	0.057 ± 0.009	0.148 ± 0.003

**Table 3:** Concentration of heavy metals in surface water of commercial farms from central coast, Bangladesh and a comparison of other relevant studies along with various standard guideline values.

Sampling Sites	Cu	Zn	Pb	Cd	Cr	Ni	Mn	References
Paikgacha (farm)	-	-	0.013	0.002	0.010	-	-	[26]
Botiaghata (farm)	-	-	0.010	0.002	0.030	-	-	[26]
Rupsha river	-	-	0.011	0.001	0.021	-	-	[26]
Gangli (farm)	-	-	0.014	0.001	0.010	-	-	[26]
Bhairab river	-	-	0.010	0.001	0.013	-	-	[26]
Satkhira (farm)	-	-	0.018	0.001	0.017	-	-	[26]
Meghna river	0.027	0.04	0.01	0.018	0.02	0.3	0.5	[27]
Edku lake, Egypt	0.17	0.08	0.21	0.01	-	-	-	[31]
Greater Noakhali (farm)	0.052	0.06	BDL	BDL	BDL	BDL	0.176	Present study
WHO	1.0	3.0	0.01	0.003	0.05	-	0.1	[28]
USEPA	1.0	1.0	0.05	0.005	0.1	-	0.05	[32]

**Table 4:** Pearson correlation matrix for three heavy metals in the water of commercial aquaculture farms of central coast, Bangladesh (significant level *p* < 0.05).

	pH	Salinity	DO	Cu	Zn	Mn
pH	1					
Salinity	0.251	1				
DO	−0.076	−0.145	1			
Cu	0.423	−0.052	−0.037	1		
Zn	−0.343	0.204	0.153	−0.279	1	
Mn	0.092	0.302	−0.015	0.066	0.270	1

**Table 5:** Principal component analysis of collected water samples of commercial aquaculture farms of the central coast, Bangladesh.

Variables	PC1	PC2
Cu	−0.571	0.717
Zn	0.858	0.006
Mn	0.544	0.743
Eigenvalues	1.357	1.066
Variance (%)	45.229	35.527
Cumulative of Variance (%)	45.229	80.756

## Data Availability

Data are provided in the article.
